# Running gait kinematics are reproducible at the beginning and end of two different intensity, energy expenditure matched runs, using either discrete points or functional data analysis

**DOI:** 10.3389/fspor.2026.1611164

**Published:** 2026-05-26

**Authors:** Sherveen Riazati, Nick Caplan, Philip R. Hayes

**Affiliations:** 1 Department of Sport and Exercise Sciences, School of Life Sciences, Northumbria University, Newcastle-upon-Tyne, United Kingdom; 2 Department of Kinesiology, San Jose State University, San Jose, CA, United States; 3 Center for Education, Research and Professional Development, Patient Care Services, Stanford Health Care, Stanford, CA, United States

**Keywords:** gait, running gait, HIIT, kinematics, reproducibility, running, functional data analysis

## Abstract

**Introduction:**

The aims of this study were to examine reliability of treadmill running kinematics: i) during two different intensity, energy expenditure matched, runs ii) at two time points within each run iii) using two different methods of statistical analysis.

**Methods:**

Twenty healthy club distance runners performed two high intensity interval runs (HIIT) and two medium intensity continuous runs (MICR). Kinematics during ground contact were analysed in all three planes of motion at the ankle, knee, and hip. Maximum angle and range of motion were identified and for Functional Data Analysis (FDA) all of the collected data was utilised. Reliability was established from both discrete points and FDA using intraclass correlation coefficient (ICC), standard error of measurement (SEM) and minimum detectable change (MDC). In addition, coefficient of variation (CV) was calculated for discrete points, however, this was not possible for FDA.

**Results:**

Both discrete point analysis and FDA showed acceptable reliability in all three planes of motion. Only three variables produced SEM values higher than 5.0 degrees while ICC values ranged from poor to excellent.

**Discussion:**

There were no differences in reliability between the start and end of the runs for HIIT or MICR. Gait kinematics during treadmill running were reliable across intensities, run type and time frames.

## 1 Introduction

A recent survey of runners found that in a year, seventy five percent of respondents experienced a running related injury (RRI) causing 50% of them to miss more than 4 days training (1). While the aetiology of overuse RRI remains unclear, there is a common consensus that risk factors e.g., excessive knee valgus or hip abduction, can be identified through biomechanical comparison of healthy and injured runners. In order to have confidence in the detection of injury risk factors through biomechanical analysis, the validity and reliability of these measures must be known (2–5).

The reliability of kinematic assessment has been overwhelmingly performed using overground running (6–8). There are, however, certain limitations when using overground running. For example, consecutive strides cannot be recorded and it requires running along a short runway, usually 10–20 m, at between ±5% to 10% of designated running speeds (9, 10). In contrast, treadmill running can provide standardised conditions with a fixed speed and motion capture calibration volume making for a more reproducible testing environment (11). Treadmill running not only has good reliability (e.g. (8, 12, 13), but also enables the recording of consecutive strides and detection of the time course of any changes (9, 11). By and large, the running speeds used in these studies tend to be absolute rather than a speed relative to the individual’s physiological capabilities. Furthermore, the reliability of kinematic data collected towards the end of a run, when athletes might be fatiguing, has yet to be established. The presumption is that reliability remains unchanged. While kinematic data collected during treadmill running has previously been used for investigating RRI, there is limited knowledge of the reproducibility or magnitude of acceptable measurement error for detecting abnormal kinematics (9, 14, 15).

Kadaba et al. (6) apart, running kinematics reliability has been through discrete points within each stride e.g., maximum or minimum joint angle, or joint range of motion (RoM) resulting in the reduction of gait cycles to a single data point. Summary biomechanical metrics derived from discrete points reduce dynamic kinematic signals and may discard essential information needed to understand the complexity of human movement. To address these limitations, raw biomechanical trajectories can be treated as smooth functions of time using functional data analysis (FDA) techniques (16), thereby preserving the full biomechanical information. Recent studies that analyse continuous biomechanical variables across the entire stride cycle are gaining popularity (17, 18). FDA is a relatively new branch of statistics, an extension of multivariate analysis, that handles statistical objects which vary continuously over a domain, for example time (16). This technique has been successfully applied to a wide range of life-science problems, including predicting maximal oxygen consumption from continuous heart-rate profiles recorded during submaximal tests (19), forecasting mortality from minute-by-minute accelerometer data (20, 21), and evaluating the therapeutic benefits of next-generation insulin drugs in diabetes (44). These examples illustrate the potential of modern statistical techniques like FDA to provide new insights by incorporating high-resolution data into the analysis.

The aim of the study was to assess the inter-day reliability of kinematic measures using FDA and discrete point methods across different time points, different exercise intensities in energy-matched treadmill runs.

## 2 Materials and methods

### 2.1 Participants

Following a power analysis and subsequent institutional ethical approval, 20 healthy, experienced, club distance runners, (*N* = 10 male; *N* = 10 female) were recruited (Table 1). The mean age of female participants was 42.2 years (± 4.0) and the mean age of male participants was 43.8 years (± 4.0). Female participants had a mean height of 164.6 cm (± 6.0) and male participants had a mean height of 181.2 cm (± 7.9). The mean mass for female participants was 58.5 kg (± 6.2) and for male participants was 77.3 kg (± 6.5). Participants were excluded if they had not participated in a timed race within two years, were not part of an affiliated running club, or had experienced any type of lower extremity injury that prevented them from running for more than a week in the past 6 months. Participants were also excluded if they had experienced any cardiovascular or neurological conditions or were allergic to adhesive material. Medical history was pre-screened via a self-reported questionnaire; eligible participants provided informed written consent prior to testing sessions.

**Table 1:** Descriptive characteristics of participants, training runs, speeds, durations, $\dot{V}O_{2}$ max, speed at lactate turnpoint (sLTP), percentage of $\dot{V}O_{2}$ max at sLTP (% $\dot{V}O_{2}$ at sLTP), represented as mean ± standard deviation.

Variables	Female	Male
	(*n* = 10)	(*n* = 10)
Age (years)	42.2 ± 4.0	43.8 ± 4
Height (cm)	164.6 ± 6.0	181.2 ± 7.9
Mass (kg)	58.5 ± 6.2	77.3 ± 6.5
HIIT Speed (m.s^−1^)	3.9 ± 0.3	4.6 ± 0.3
HIIT rep duration (min:sec)	03:24 ± 13(s)	02:47 ± 16(s)
MICR Speed (m.s^−1^)	3.3 ± 0.2	3.6 ± 0.4
MICR duration (min:sec)	32:15 ± 02:01	25:53 ± 03:40
$\dot{V}O_{2}$ max (ml.kg^−1^.min^−1^)	53.6 ± 5.4	60.5 ± 4.4
sLTP (m.s^−1^)	3.3 ± 0.2	3.7 ± 0.4
% $\dot{V}O_{2}$ max at LTP	81.4 ± 5.5	72.7 ± 8.1

### 2.2 Procedure

**Figure 1:**
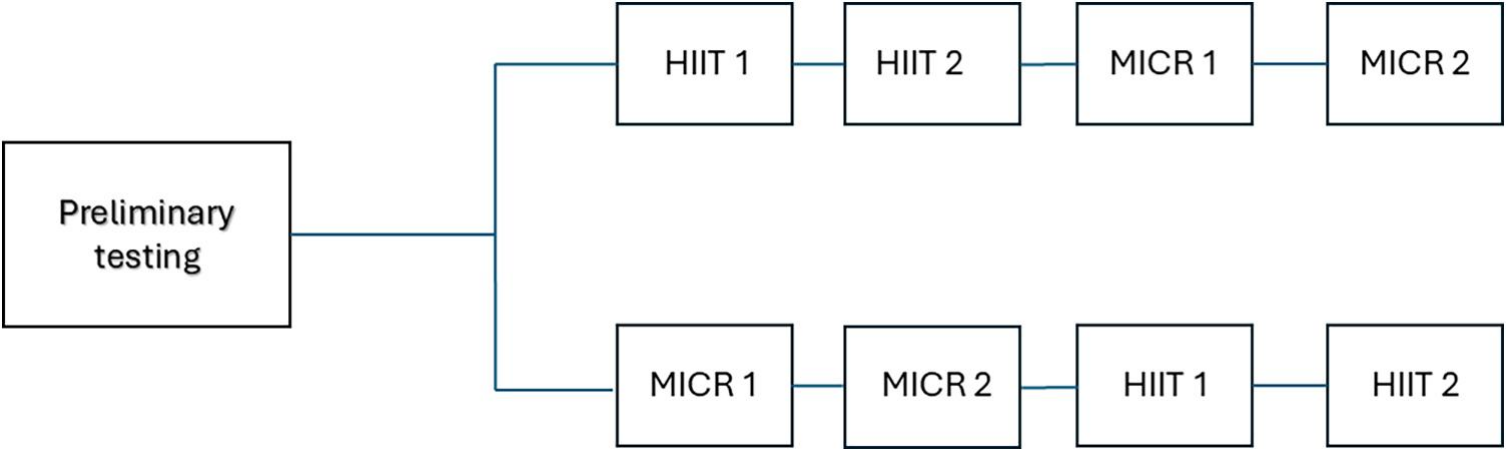
Study design.

Participants completed five sessions, including a preliminary session to collect physiological data used to set running speeds and durations of the reliability trials. This was followed by four reliability trials, two high intensity interval training (HIIT) and two medium intensity continuous runs (MICR) (See Figure 1). All tests were conducted at the same time of day to minimize diurnal variation (22). Participants were asked to wear their preferred, same, footwear throughout and to self-report their habitual dietary regimen, while refraining from high volume or intensity training 48 h prior to testing.

#### 2.2.1 Preliminary testing

Initial measurements of mass, height and kinanthropometric measures were taken according to Society for the Advancement of Kinanthropometry (ISAK) guidelines by an ISAK qualified practitioner. Participants completed an incremental treadmill (ELG2, Woodway, Germany; belt length 2.59 m, belt width 1.12 m) test to determine maximum steady state and $\dot{V}O_{2}$ max. Expired gas analysis was measured by Cortex Metalyser 3B (Leipzig, Germany), calibrated according to manufacturer’s instructions prior to each test. A 5-minute warm-up run was completed to familiarise participants with the treadmill and equipment used for expired gas collections.

The sub-maximal test consisted of a series of incremental 4-minute stages at 0% gradient, separated by 60-s recovery (23). Stages increased by 1 km.h^−1^ until lactate turn point (LTP) had been exceeded, with the initial speed set according to the participant’s current performance level. Between stages, a fingertip capillary blood sample was taken for analysis of blood lactate concentration (Biosen C-line, EKF diagnostics, Germany). Following a 15-min recovery, participants completed a $\dot{V}O_{2}$ max test with initial speed set at 4 km.h^−1^ under the speed at LTP (sLTP) at a 0% gradient. The treadmill speed was increased by 0.5 km.h^−1^ every 30 s until volitional exhaustion occurred.

#### 2.2.2 Reliability trials

Reliability of gait parameters was assessed using two different types of run; high intensity interval training and medium intensity continuous running. The duration and speed of each run was individualised based on $\dot{V}O_{2}$ max and LTP. The HIIT session was a modification of the James and Doust (24) protocol that has previously been shown to cause fatigue. It consisted of six repetitions of 800 meters, run at 1 km.h^−1^ below the speed at $\dot{V}O_{2}$ max ($s\dot{V}O_{2}$ max), with a 1:1 work: rest ratio. The rest was active, with participants walking at 4 km.h^−1^. The MICR was run at a speed halfway between the speed at lactate threshold (*s*LT) and *s*LTP; the duration set to ensure matched energy expenditure (EE) with the HIIT session. A minimum of 72 h elapsed between each session to ensure no residual fatigue effects (24).

Briefly, the $\dot{V}O_{2}$—running speed relationship was established using the expired gas data from the final 60s of each sub-maximal stage. From this relationship EE (kJ.min^−1^), for both the speed of the HIIT intervals and recoveries, was calculated using the method of Shaw et al. (25). Both EE values were multiplied by the total time spent at each speed and then summed to give the total EE. The total EE from the HIIT session was divided by the rate of EE for the MICR speed to determine the MICR duration.

### 2.3 Kinematic analysis

Running kinematics were captured via a 14-camera 3-dimensional kinematic analysis system (MX; Vicon Motion Systems Ltd., Oxford, England) sampling at 500 Hz (26). Marker trajectories were recorded for 25 s at the end of the first minute and the final minute of each run. Retroreflective markers were placed bilaterally on the anterior superior iliac spine (ASIS), posterior superior iliac spine (PSIS), thigh, condyles of the femur, lateral shank, lateral malleoli, base of the 2nd metatarsal, and calcaneus, according to the lower body Plug-in-Gait (PiG) model. The markers were carefully placed, by the same researcher, on the desired landmarks with double-sided tape and the surrounding base was also taped down with double sided tape. The area of the placement for thigh, knee and shank were shaved if required. Wand markers were used for the thigh and shank in order to obtain rotational movements of the joints. Wand markers for the lateral thigh were placed 2/3 length of the femur, measured from the greater trochanter to lateral epicondyle of the femur. Wand markers for the shank were placed 2/3 of the shank, measured from fibular head to apex of lateral malleoli. Participants were given compression leggings to wear in order to improve marker adhesion. Hip markers were taped around the hip using soft adhesive tape to avoid impeding hip movement and to ensure they remained in place throughout the run.

### 2.4 Data analysis

Marker trajectories were filtered using a fourth order Butterworth filter with a 6 Hz cut-off frequency. Gait identification was achieved through manual detection of foot strike and foot off for 30 consecutive strides through visual inspection of the heel markers in the sagittal plane (27). Maximum angle and range of motion of the ankle, knee, and hip in the sagittal, frontal and transverse planes during stance phase were exported. All kinematic data were processed using a custom written script in Matlab (2018a, The Mathworks, Inc. Natick, MA, USA).

### 2.5 Statistical analysis

#### 2.5.1 Relative reliability

Relative reliability was reported through intraclass correlation coefficients for repeated measures (ICC (3,1)) for both discrete and FDA analysis. An ICC of <0.50 was considered poor, while between 0.5 and 0.75 was considered moderate and between 0.75 and 0.90 was considered good, with excellent reliability >0.90 (28).

#### 2.5.2 Absolute reliability

Absolute reliability was expressed as standard error of measurement (SEM), representing a combination random and systematic error (29), expressed as.

SEM=SD(√(1−ICC)) (1)

where SD denotes standard deviation of all scores. Minimum detectable change (MDC) (Equation 2) was calculated to estimate the minimum amount of change needed to be 95% confident that a real change had occurred and calculated as

MDC=SEM×1.96×√2 (2)

Coefficient of variation (Equation 3) could only be calculated for discrete point assessments and not for FDA and calculated as

CV=(SD/mean)×100 (3)

#### 2.5.3 Functional data analysis reliability

To apply FDA effectively, the data curves for each ground contact were aligned using the approach of (30). The intraclass correlation was calculated using approach of Di et al. (31). Standard error of measurement was calculated according to equation (1) by replacing standard deviation with a functional mean standard deviation. All FDA analyses were performed using R statistical software (R Development Core Team, 2012) using packages and refund packages available through FDA.usc (32).

## 3 Results

### 3.1 Relative reliability

In general, the study produced poor to excellent ICCs (0.46–0.94) during discrete traditional assessment for all three planes of motion and poor to good ICCs (0.04–0.84) for all three planes of motion when using FDA (Tables 2, 3). Functional data analysis produced lower ICCs (Table 3) in MICR compared to HIIT for all three joints in the sagittal plane and for the ankle in the frontal plane. When using traditional methods, RoM produced better reliability for the knee and hip compared to maximum angle in all planes of movement (Table 2). In FDA, the only discrepancy between the three planes was observed at the knee joint, where the sagittal plane produced the lowest ICCs.

**Table 2:** Comparison of intraclass correlation coefficients (ICC), standard error of measurement (SEM), Minimum detectable changes (MDC), for maximum angles (Max) and range of motion (RoM) of stance phase in sagittal and frontal planes of motion for ankle, knee, and Hip in HIIT start and end; and MICR start and end.

	Sagittal	ICC	SEM	MDC	Frontal	ICC	SEM	MDC	Transverse	ICC	SEM	MDC
Ankle Max	HIIT start	0.85	3.3	9.1	HIIT start	0.84	2.0	5.5	HIIT start	0.90	5.1	14.1
	HIIT end	0.84	3.4	9.4	HIIT end	0.82	2.7	7.5	HIIT end	0.94	3.8	10.5
	MICR start	0.79	3.5	9.7	MICR start	0.71	1.8	5.0	MICR start	0.83	3.1	8.6
	MICR end	0.81	3.5	9.7	MICR end	0.75	2.1	5.8	MICR end	0.88	4.6	12.8
Ankle RoM	HIIT start	0.67	3.7	10.3	HIIT start	0.49	1.3	3.6	HIIT start	0.79	1.8	5.0
	HIIT end	0.78	3.5	9.7	HIIT end	0.64	1.3	3.6	HIIT end	0.77	2.5	6.9
	MICR start	0.61	3.6	10.0	MICR start	0.70	1.0	2.8	MICR start	0.78	2.0	5.5
	MICR end	0.77	2.5	6.9	MICR end	0.54	1.1	3.0	MICR end	0.70	2.4	6.7
Knee Max	HIIT start	0.90	2.0	5.5	HIIT start	0.50	3.0	8.3	HIIT start	0.88	4.0	11.1
	HIIT end	0.80	2.9	8.0	HIIT end	0.46	5.0	13.9	HIIT end	0.86	4.7	13.0
	MICR start	0.91	2.0	5.5	MICR start	0.86	2.7	7.5	MICR start	0.83	4.5	12.5
	MICR end	0.74	3.7	10.3	MICR end	0.82	3.7	10.3	MICR end	0.83	4.2	11.6
Knee RoM	HIIT start	0.92	1.7	4.7	HIIT start	0.47	1.6	4.4	HIIT start	0.63	2.9	8.0
	HIIT end	0.81	2.5	6.9	HIIT end	0.68	1.3	3.6	HIIT end	0.81	2.6	7.2
	MICR start	0.94	1.5	4.2	MICR start	0.57	2.4	6.7	MICR start	0.63	3.4	9.4
	MICR end	0.94	1.9	5.3	MICR end	0.75	1.4	3.9	MICR end	0.63	3.3	9.1
Hip Max	HIIT start	0.85	2.9	8.0	HIIT start	0.74	1.8	5.0	HIIT start	0.82	4.8	13.3
	HIIT end	0.88	2.6	7.2	HIIT end	0.73	2.3	6.4	HIIT end	0.79	4.8	13.3
	MICR start	0.88	2.9	8.0	MICR start	0.73	1.0	2.8	MICR start	0.91	4.8	13.3
	MICR end	0.72	3.6	10.0	MICR end	0.75	1.0	2.8	MICR end	0.86	5.7	15.8
Hip RoM	HIIT start	0.74	1.9	5.3	HIIT start	0.76	1.2	3.3	HIIT start	0.61	2.6	7.2
	HIIT end	0.88	1.6	4.4	HIIT end	0.78	2.1	5.8	HIIT end	0.53	3.3	9.1
	MICR start	0.86	1.4	3.9	MICR start	0.78	2.0	5.5	MICR start	0.46	4.6	12.8
	MICR end	0.88	1.5	4.2	MICR end	0.93	1.2	3.3	MICR end	0.66	2.8	7.8

**Table 3:** FDA comparison of intraclass correlation coefficients (ICC); standard error of measurement (SEM), Minimum detectable changes (MDC) of stance phase in sagittal, frontal, and transverse planes of motion for ankle, knee, and Hip in HIIT start and end; and MICR start and end.

	Sagittal	ICC	SEM	MDC	Frontal	ICC	SEM	MDC	Transverse	ICC	SEM	MDC
Ankle	HIIT start	0.82	0.9	2.5	HIIT start	0.81	0.9	2.5	HIIT start	0.76	1.9	5.3
	HIIT end	0.58	1.3	3.6	HIIT end	0.58	1.3	3.6	HIIT end	0.48	2.7	7.5
	MICR start	0.26	1.6	4.4	MICR start	0.26	1.6	4.4	MICR start	0.70	1.8	5.0
	MICR end	0.17	1.9	5.3	MICR end	0.22	1.6	4.4	MICR end	0.64	1.9	5.3
Knee	HIIT start	0.66	1.3	3.6	HIIT start	0.37	1.7	4.7	HIIT start	0.63	2.0	5.5
	HIIT end	0.52	1.6	4.4	HIIT end	0.50	1.7	4.7	HIIT end	0.51	2.3	6.4
	MICR start	0.50	1.7	4.7	MICR start	0.79	1.2	3.3	MICR start	0.48	2.4	6.7
	MICR end	0.04	2.6	7.2	MICR end	0.73	1.4	3.9	MICR end	0.52	2.3	6.4
Hip	HIIT start	0.72	1.3	3.6	HIIT start	0.74	0.9	2.5	HIIT start	0.56	2.1	5.8
	HIIT end	0.68	1.4	3.9	HIIT end	0.72	1.0	2.8	HIIT end	0.40	2.5	6.9
	MICR start	0.53	1.7	4.7	MICR start	0.80	0.8	2.2	MICR start	0.70	2.0	5.5
	MICR end	0.24	2.2	6.1	MICR end	0.70	1.0	2.8	MICR end	0.66	2.0	5.5

Within joints and planes of motions, there were limited differences in ICC values when comparing the start or end of either run intensity. Functional data analysis produced the only discrepancy; at the end of the run ICC values in the sagittal plane ranged from 0.04–0.68 compared to 0.26–0.82 at the start (Table 3). For discrete point analysis each plane of movement produced similar ranges of ICC values at the start and end of the runs.

### 3.2 Absolute reliability

**Figure 2:**
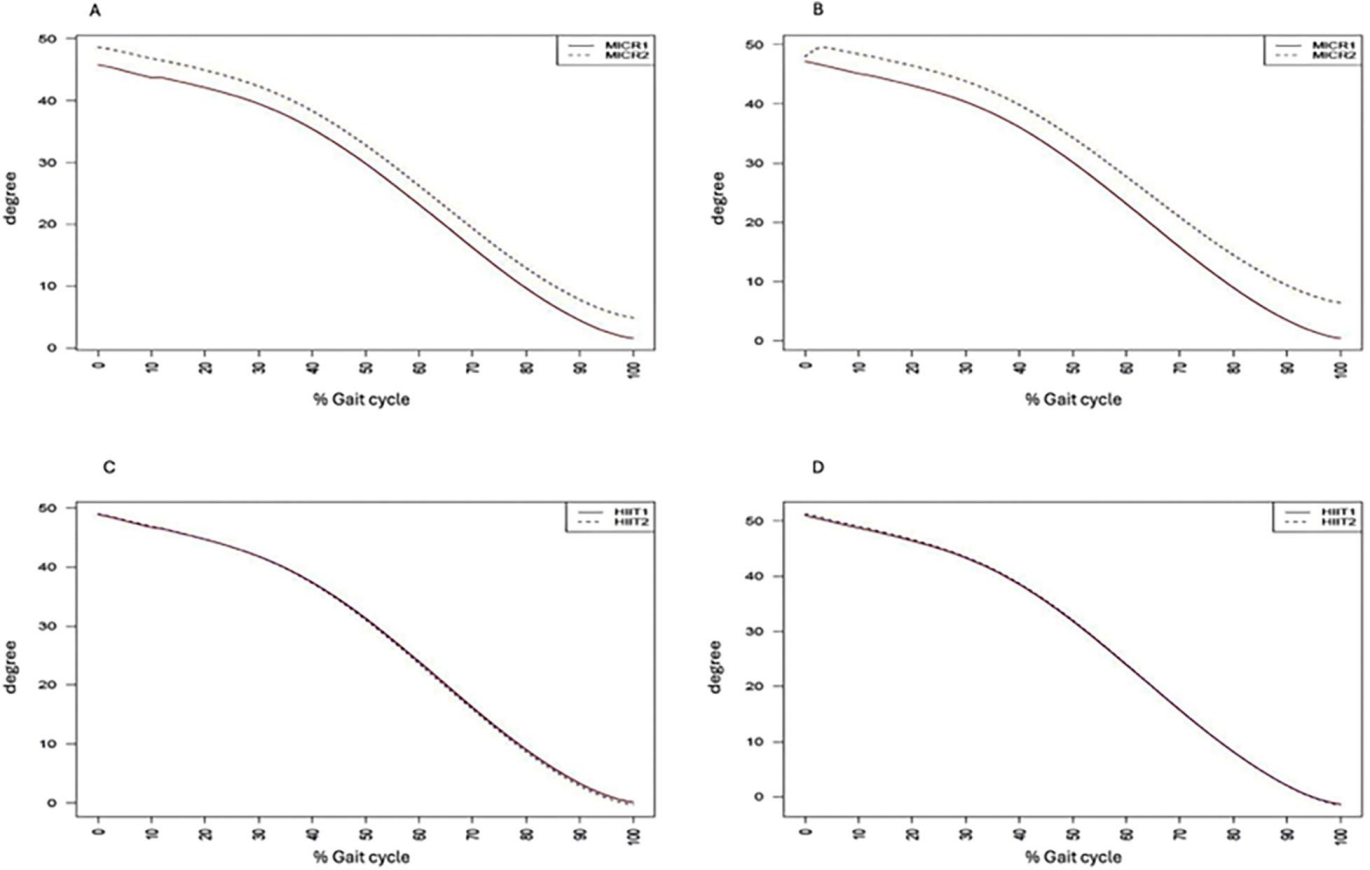
FDA examination of Hip sagittal plane. **(A)** MCR start. **(B)** MICR end. **(C)** HIIT start and **(D)** HIIT end.

In discrete measurements, the SEM ranged from 1.0° to 5.5°, with the highest values observed in transverse planes of motion (1.8°–5.7°), while sagittal (1.4°–3.7°) and frontal planes (1.0°–5.0°) produced similar values. There were no differences observed between HIIT and MICR for SEM for either RoM or maximum angles, in the sagittal or frontal planes. Maximum angles for the transverse planes of motion produced the highest SEM values of the three planes. Functional data analysis produced slightly lower SEMs for all three joints and planes, as well as for both MICR and HIIT (0.8°–2.7°) (see Table 3 and Figure 2).

The comparison between the start and end of the runs found similar SEM values, this was consistent across run type and plane of movement (see Tables 2, 3). Examination of the coefficient of variation found no differences between MICR and HIIT, or between time points for all joints and planes. Within the sagittal plane, CV values ranged from 2.2% to 6.8% and between 2.3% and 13.9% within the frontal plane. RoM values for the knee joint produced the highest CV. The transverse plane produced the highest CV of the three planes for the maximum angles of both ankle and hip, with a range from 3.0% to 15.9%, with ankle_max_ and both hip_max_ and RoM producing the highest values.

## 4 Discussion

The aim of this study was to determine the between-day reliability and measurement error in kinematic assessment during treadmill running for two different running speeds, at both the start and end of each run. In addition to the traditional discrete point analysis, functional data analysis was undertaken to examine kinematic reliability and measurement error. The results of this study demonstrate good to excellent reliability for kinematic assessment during treadmill running. There were minimal differences in the reliability of the two-energy expenditure matched running speeds even though they differed in physiological demand. Furthermore, regardless of time point of data collection, the reliability of the capture remained good to acceptable. The use of functional data analysis provided a slight reduction in measurement error compared discrete point analysis.

Generally, the SEMs in this study were within the range typically found in other reliability studies (7, 8, 12, 33, 34). Furthermore, like most previous studies, we found transverse plane reliability to be poorer than frontal or sagittal plane measures. Like Bramah et al., and Noehren et al., we recorded gait kinematics during treadmill running, to enable the capture of consecutive strides, rather than a compilation of individual strides. However, we captured 30 foot contacts, by comparison, most previous reliability studies that have tended to use 5–10 contacts, based on the recommendations of Riazati et al. (27). Our SEM values for peak joint angles and range of movement, at the beginning of both MICR and HIIT, tended to be higher than Bramah et al. in all variables except hip and knee sagittal plane RoM. Compared to Noehren et al., there was no clear pattern with scores being similar, higher and lower to their manual marker placement condition. Inconsistencies in marker placement account for much of the day-to-day variance in scores (8), so too can training status, with higher caliber runners exhibiting more stable spatiotemporal variables (35, 36).

This study concurs with the views of both McGinley et al. (37) and Bramah et al. (12) that reliability studies should provide SEM values of absolute reliability, allowing the determination of MDC. SEM is the expected error within a measurement 67% of the time, while MDC is the amount of change required to be sure that the change is real, rather than due to measurement error. McGinley et al. (37) went further, suggesting that SEM values beyond 5° could ‘mislead clinical interpretation’. All bar three (transverse peak angle at the ankle and hip, frontal peak knee angle) of our SEM values were below 5°, however these three did result in MDC values, for discrete points, of as high as 15.8° (see Table 2). Two of these large MDC values (peak knee and hip angles) occurred at the end of exercise and could therefore be affected by fatigue. In agreement with both McGinley et al. (37), Bramah et al. (12), we concur that values of this magnitude are too high to distinguish between non-injured runners and those who are injured, have a clinical condition or to assess the effectiveness of interventions to alter gait.

Numerous studies have used an exhaustive run to examine the effect of fatigue on gait kinematics or differentiate between injured and non-injured runners [e.g., (38, 39)]. The underlying assumption here is that the reliability of gait kinematics at the end of the run is unaffected by fatigue. Martens et al. (40) examined the fatigue response to spatiotemporal stride parameters, finding good to moderate reproducibility during swing phase but poor reproducibility during the stance phase. To the best of our knowledge, this study is the first to examine not only the reproducibility of gait kinematics at the end of a run, but in two different types of run. We found no consistent pattern of change in any variable or plane of motion between the beginning and end of either run type, indicating changes in kinematic variables with fatigue are reproducible irrespective of intensity. Furthermore, there were no clear differences between MICR and HIIT, this could be due to matching them according to energy expenditure or that they were not completed to exhaustion. Further research is required to elucidate if there is a difference in kinematics at the point of exhaustion, rather than when runners are fatigued.

This was the first study to quantify measurement error during the entire ground contact phase by using FDA. Similar SEM values were attained, despite the greater degrees of freedom, demonstrating that FDA is an appropriate tool to use with kinematic data (see Figure 2). Our SEM values with FDA were 2.7° or less, with MDC values below 7.2°. The decrease in absolute measurement error from using FDA could improve the sensitivity of gait analysis in differentiating between sub-groups or evaluating the effectiveness of an intervention. While absolute reliability improved with FDA, relative reliability measured using ICC was poorer. Giraud (41) suggested this could be due to the increased data set being more likely to contain outliers. From a practical perspective, the improvement in SEM with FDA is of considerable importance showing a reduction in the likely measurement error practitioners and researchers can expect (29). The ICC value however, while providing an indicator of consistency, is not particularly useful in interpreting human movement (42). Both Olds (42) and Weir (29) consider SEM to be of more practical value than ICC.

The low SEM values in both discrete point and FDA assessments suggests that the approaches taken in this study were successful in producing reliable results for kinematic assessment during treadmill running. Marker placement along with skin and soft tissue artefacts have been linked as large sources of errors within kinematic analysis (7, 8). A 10 mm displacement can cause up to 7.6° of change in the ankle and up to 6° in the knee (43). The low measurement error and variability suggest that the approaches taken in this study were successful in minimising this.

## 5 Conclusion

This was the first study to examine the reliability of treadmill running kinematics using functional data analysis. Both FDA and the more traditional discrete point approach demonstrated good reliability in the sagittal and frontal planes. Like previous studies, traverse plane reliability was poorer for discrete point analysis, however FDA provided good reliability. Two further novel findings of this study were that reliability was compared across two different intensity runs and at the beginning and end of those runs. These results support the use of treadmill-based kinematic assessments in both clinical and performance settings and encourage the use of FDA in future reliability studies

## Data Availability

The raw data supporting the conclusions of this article will be made available by the authors, without undue reservation.
